# New Records of Mosquitoes (Diptera: Culicidae) from Bolívar State in South Eastern Venezuela, with 27 New Species for the State and 5 of Them New in the Country

**DOI:** 10.3389/fpubh.2014.00268

**Published:** 2015-03-13

**Authors:** Jesús Berti, Hernán Guzmán, Yarys Estrada, Rodrigo Ramírez

**Affiliations:** ^1^Laboratory of Entomology, Center for Endemic Diseases Studies, Maracay, Venezuela

**Keywords:** mosquitoes, vectors, inventory, emerging diseases, Bolívar State, Gran Sabana, Venezuela

## Abstract

This is the first part of a series of studies related to mosquito ecological and biogeographic aspects. A total of 69 mosquito species (Diptera: Culicidae) was collected in 16 localities sampled in the Gran Sabana Municipality, Canaima National Park, and Venezuela. Twenty-seven mosquito species are recorded for the first time from Bolívar State, Venezuela. Five of them species are reported for the first time in Venezuela: *Anopheles malefactor* Dyar and Knab (1907); *Chagasia bonneae* Root (1927); *Chagasia ablusa* Harbach (2009); *Culex anduzei* Lane (1944), and *Uranotaenia leucoptera* Theobald (1907). Their medical importance is commented, and ecological and epidemiological aspects are discussed. A checklist of the mosquito species reported in the Gran Sabana County is given.

## Introduction

The occupation of Amazonia in Brazil and Venezuela, has formed part of the integration processes that both governments are implementing in their common border, which makes evident that both governments are aware of the importance that the Amazonia has in our present world (1). Diseases such as malaria, dengue, Chikungunya, Yellow Fever, Mayaro virus, West Nile virus, and several emerging and reemerging arboviruses, which are responsible for millions of cases of sickness and death among people living in the tropical regions, continue to be of great concern to the World Health Organization authorities in our present world (2). Dengue is an important arbovirus that affects humans; it is transmitted by *Aedes aegypti* (Linnaeus) and *Aedes albopictus* Skuse. *A. aegypti* and *A. albopictus* species have been involved in Dengue transmission in the Manaus rural areas, Brazilian Amazon (3). *A. albopictus* a secondary dengue vector in Asia, has spread to America and Europe largely, due to the international trade of used tires (a typical larval habitat), timber, and other goods such as “lucky bamboo” (a decorative house plant that is marketed worldwide). This species has a wide geographical distribution; it is particularly resistant, and can survive in both rural and urban environments. Mosquito’s eggs are highly resistant and can remain viable throughout dry season, and can survive in cold temperature regions of Europe (3). *A. albopictus* and *A. terrens* (Walker) species are two potential Yellow Fever vectors in jungle. These species utilize a wide variety of natural larval microhabitats, such as tree-holes, bamboo internodes, and artificial containers and may be found in the same natural environments as *Haemagogus* species (1). *Haemagogus* species have been involved in Sylvain yellow fever transmission in Venezuela (4). Human malaria, is one of the most serious parasitic diseases in tropical ecosystems, it is caused by parasites of the genus *Plasmodium* (Apicomplexa: Plasmodidae) and transmitted among human hosts by bites of the infected *Anopheles* female (1). Five parasite species cause malaria in humans. *Plasmodium falciparum* and *P. vivax* are the two most common. *P. falciparum* is the most dangerous, with the highest rates of mortality (1).

In 2012, Venezuela reported the highest recorded incidence of malaria in its history with 51,264 cases. In Bolívar State, incidence cases increased to 44,180 (86.2% of the country); with three counties (Sifontes, Gran Sabana, and Cedeño) in “epidemic” and two counties (Piar and Sucre) in “alarm” status (5). *Anopheles darlingi* Root has been considered as human malaria’s principal vector in South America. In Amazonas and Bolívar states, it is responsible for 90% of malaria cases reported in Venezuela (1, 6).

Mosquito borne diseases such as malaria, dengue, Venezuelan Equine Encephalitis (VEE), West Nile virus and others equine encephalitis, Mayaro, or Chikungunya are zoonoses with increasing incidence in the current decade in tropical and temperate countries. These diseases emerge as a consequence of changes made to terrains that often increase the natural and artificial mosquito larval habitats. Many of these mosquito species are of public health importance. Mosquito’s population increases result in a risk increased of tropical diseases transmission (1). There are many factors that can accelerate the emergence of zoonoses, such as environmental changes, habitat modifications, variations of human and animal demography, deterioration of strategies of vector control, or changes in pathogen genetics (1). Efforts to control such species that transmit emerging diseases have primarily been concentrated on the use of synthetic insecticides. Unfortunately, this has resulted in the appearance of physiological mosquito resistance, toxicity problems to human, environmental contamination, ecological imbalance, and economic burden (1). Such problems have created the need to look for alternative, environmentally friendly control mechanisms, based on those found in nature. These include the essential oils from plants, some of which have been used by people for medicinal purposes; also biological control (e.g., *Bacillus sphaericus* against *Anopheles*) and biochemical control with synthetics juvenile hormones (7, 8).

It is well known that the rate of species lost worldwide surpasses that by which taxonomic knowledge is increased (9). Thus, it is necessary to intensify studies focusing on diversity and from areas with an acceptable level of conservation (e.g., Canaima National Park, Venezuela). The Gran Sabana Region (Canaima National Park) is an undulating plain grass-dominated upland savanna covering close to 18,000 km^2^, with altitudes ranging from 750 to 1,450 m (10). Most of the Gran Sabana uplands have a humid submontane climate, with average annual temperatures ranging between 18 and 24°C, average annual rainfall between 2,000 and 3,000 mm, and a short dry season occurring from December to March. This area is drained by tributaries of the Orinoco River (Venezuelan part of the igneous metamorphic Guyana Shield), most of them black-water Rivers, with very acidic and low mineral waters (10). The ecological studies in the Gran Sabana are relatively scarce, especially for short and long-term evaluations on possible changes induced by human activities (e.g., climatic change). Additionally, ecological studies on richness and distribution at regional levels are few. It is well known that Culicidae larvae are dependent on habitat characteristics and that they are sensitive to biotic factors, as predators and also to several abiotic factors (pH, temperature, dissolved oxygen, salinity, and conductivity).

The present article, refers to the finding of 69 species and 17 genera of mosquito (Diptera: Culicidae), collected in the Gran Sabana county, Bolívar State, Venezuela. Twenty-seven mosquito species are recorded for the first time from Bolívar State, and five species are reported for the first time in the country.

## Materials and Methods

### Study area

**Figure 1:**
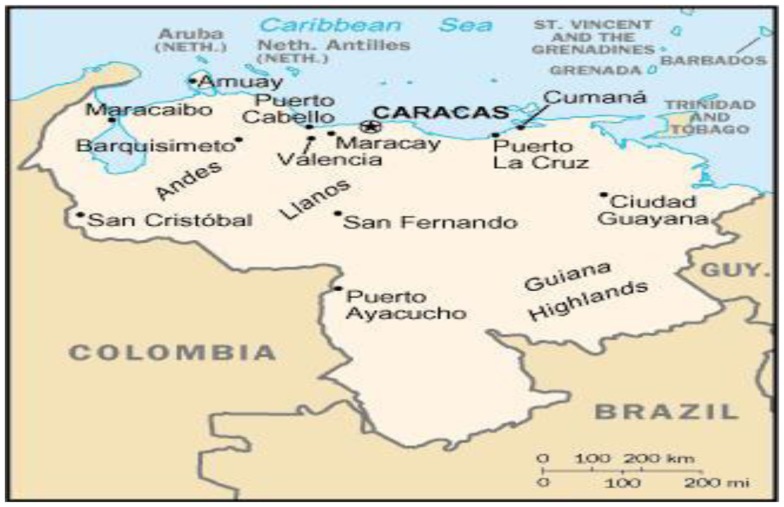
**Geographic situation of the Guiana Highlands in Venezuela**.

**Figure 2:**
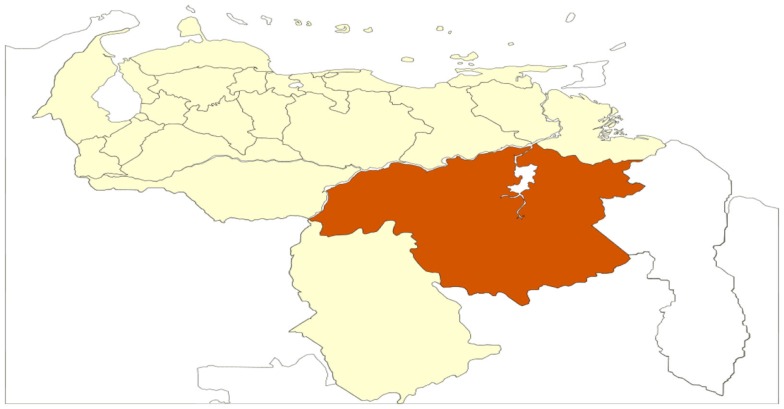
**Relative situations of the Bolivar State in Venezuela**.

**Figure 3:**
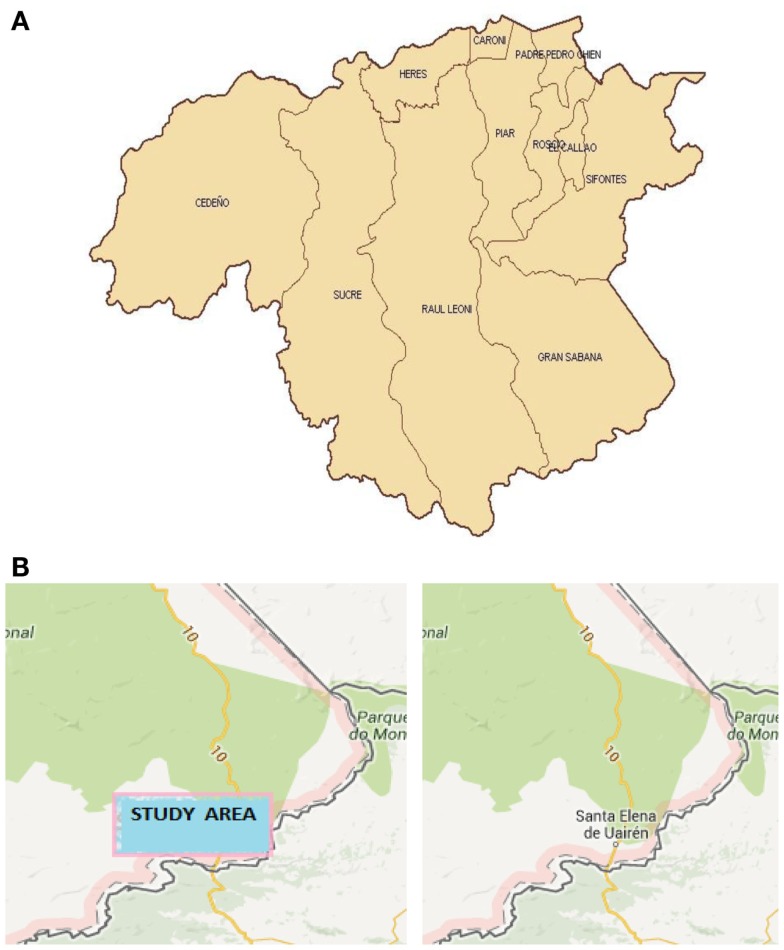
**(A)** Relative situation of the municipalities in the Bolivar State; and **(B)** situations of the study area and Santa Elena of Uairén.

The study area is located in the southern of Gran Sabana Municipality, Canaima National Park, a natural protected area in southeastern Venezuela, Amazonian Region that borders Brazil and Guyana (Figures 1–3). This study is based on material collected in indigenous territory (Pemón ethnic group) of Gran Sabana, Canaima National Park, located in Bolívar State, and roughly occupies the same area as the Gran Sabana region (11). The park was established on 12 June 1962. It is the second largest park in the country, after Parima-Tapirapecó in Amazonas State, and sixth biggest national park in the world (1). About 65% of the park is occupied by plateaus of rock called tepuis, which are a kind of plateau of 1.7–1.8 billion years old. The oldest Roraima sandstone is estimated to be 1.6 billion years old. These ancient mountains of the Guiana Shield (Figure 1) in the Amazonian Region (12) constitute a unique biological environment and are of great geological interest, which makes them one of the oldest formations in the world (11).

The park is home to indigenous Pemón Indians (Pemón ethnic group), part of the Carib linguistic group (11). Pemón Indians have an intimate relationship with the tepuis, and believe they are the home of the “Mawari” spirits. Most transport within the park is done by light plane, from airstrips built by various Capuchin missions, or by foot and canoe. Pemón indigenous have developed some basic and luxurious camps, which are mainly visited by tourists from around the world and Venezuelan tourists. In 1994, Canaima National Park was named a World Heritage Site by UNESCO, as a natural reserve that has abrupt relief special and unique around the world, “The Tepuis,” The park includes the entire watershed of the right bank of the Caroní River, and two of the highest waterfalls in the world, the Angel Falls and the Kukenán Falls and plenty of waterfalls of lower altitude (11).

Field surveys of mosquito adults (human bait) and mosquito larvae sampling, were carried out in the Gran Sabana County. The annual mean temperature is 22°C (18–24°C); the total annual rainfall is 1,500–5,700 mm, with annual average rainfall between 2,000 and 3,000 mm, with altitudes ranging from 750 to 1,450 m and annual mean of 205 days with rain per year, and a very short dry season occurring from December to March (10). The estimated population is 39,000, mainly concentrated in the capital, Santa Elena of Uairén (Figures 2 and 3).

Sixteen localities and four rivers were sampled for mosquitoes in the Gran Sabana County near the common border Brazil–Venezuela, between August 2011 and November 2013. These sites were selected as representative areas of the Gran Sabana, Venezuelan Canaima National Park. All localities are at altitudes >600 m. These altitudes are variables and can range from 600 to 1,450 m. These localities and rivers are: Santa Elena de Uairén, Waramasén, Manak-Krú, Maurak, Colinas de la Laguna, Altamira, San Antonio, Kinok-Pon-Parú, La Primavera, Chiricayén, Chiririka, Uaiparú, Betania, Kamaiwa, El Paraíso, El Paují, Chiririka, Uairén, Uaiparú, and Kukenán rivers.

### Collection and identification of mosquitoes

**Figure 4:**
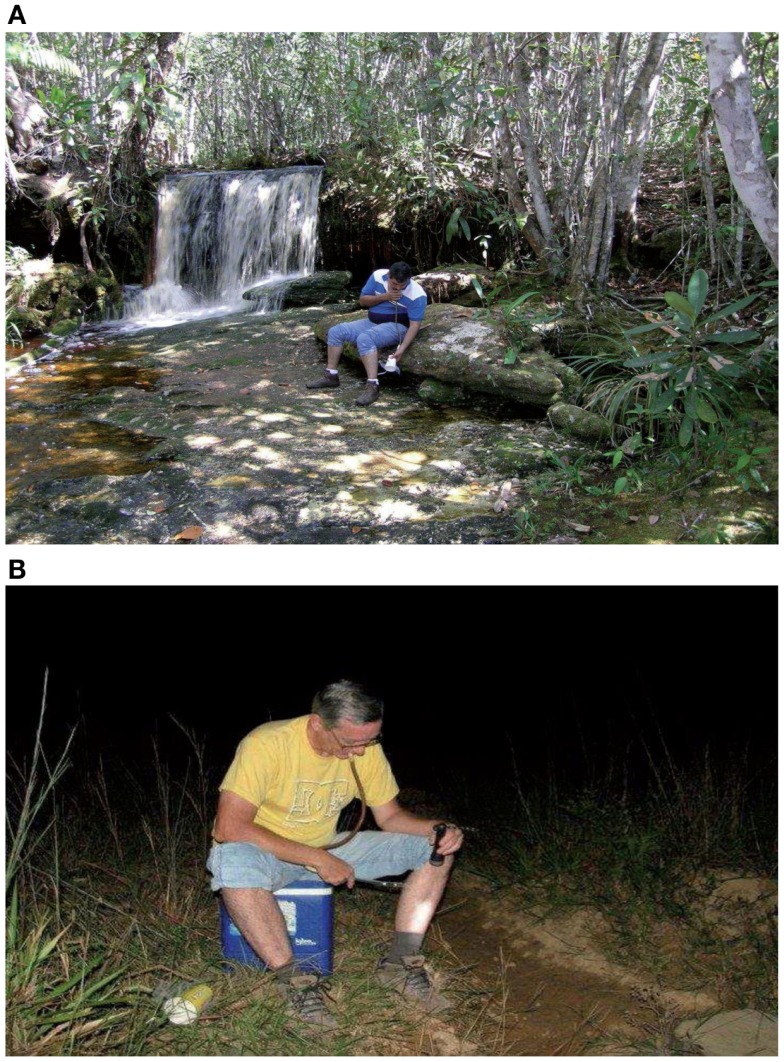
**(A)** Adult collections on human landing catch during the day. **(B)** Adult collections on human landing catch in streams edges during the nights.

A sampling program of mosquito larvae was carried out between August 2011 and November 2013 by visiting the study area periodically (dry and rainy season every year). Mosquito samples were taken with a standard dipper. The natural aquatic habitats (not-Phytotelmata) were *a priori* classified into four categories: lagoons, streams, rivers, and freshwater herbaceous swamps. At each breeding site, 30 dips for mosquito larvae samples were made. Most of the mosquito collections were made in tropical humid forests and edges of streams, rivers, and herbaceous swamps (morichales) of savanna areas in Gran Sabana County. Additionally, mosquito larvae were collected in artificial and natural containers. In Phytotelmata, larvae were collected by extracting water with a plastic pipettes from tree-holes, cut bamboo internodes, leaf axils of bromeliads, foliar axils of Araceae, fallen leaves of Musaceae, fallen palm spates, especially the “Moriches” palms (*Mauritia flexuosa*), and from floral bracts of Heliconiaceae. Immature mosquitoes were collected from 40 samples (water-holding structures) per plant species per locality. Collected immature specimens (IV instars larvae) from half the samples (20 samples) were preserved in ethanol (90%) for identification purposes. Specimens (larvae and pupae) from the rest of samples (50%) were pooled, transported, and reared in the field laboratory to obtain the associated specimens. The species presents, were recognized on the basis of correlated anatomical features in associated life stages. Sampling to adult collections on human landing catches (Figures 4A,B) were carried out in the same localities and rivers. The taxonomic determination of the specimens was based on direct observation of morphological characters, through a stereoscopic microscope (adults) and transmitted light microscope (larvae), using several taxonomic keys and descriptions and re-descriptions of species. The abbreviations employed for mosquito genera and subgenera are those proposed by Reinert (13).

All diagnostic and differential characters were confirmed by using several taxonomic keys and using descriptions and re-descriptions of species (4, 14–28, 29). All immature and adults specimens are deposited at the Collection of Center for Endemic Disease studies, located in Las Delicias, Maracay, Venezuela.

## Results

A list of mosquito taxa of the Gran Sabana Municipality is presented. A total of 69 mosquito species (Diptera: Culicidae) and 17 mosquito genera from the indigenous territory (Pemón ethnic group) are reported (Table 1). A total of 19 species of anophelines were collected; 17 of them belonging to genus *Anopheles* Meigen and 2 species belonging to genus *Chagasia* Cruz. Additionally, 50 species of culicines were collected, belonging to 14 genera of *Culicinae* and one genus (*Toxorhynchites*) of *Toxorhynchitinae* (Table 1).

**Table 1:** **Inventory of Culicidae from Gran Sabana Municipality, Venezuela**.

**Culicidae: 1. Subfamily Anophelinae**
1.1 *Anopheles* (Human Malaria vectors)
*Anopheles (Nyssorhynchus) triannulatus* Neiva and Pinto (1922)
*Anopheles (Nyssorhynchus) brasiliensis* Chagas (1907)
*Anopheles (Nyssorhynchus) marajoara* Galvao and Damasceno (1942)
*Anopheles (Nyssorhynchus) nuneztovari* Gabaldon (1940)
*Anopheles (Nyssorhynchus) argyritarsis* Robineau-Desvoidy (1827)
*Anopheles (Nyssorhynchus) darlingi* Root (1926)
*Anopheles (Nyssorhynchus) oswaldoi* Peryass (1922)
*Anopheles (Nyssorhynchus) strodei* Root (1926)
*Anopheles (Nyssorhynchus) rangeli* Cova-García and López (1940)
*Anopheles (Lophopodomyia) squamifemur* Antunes (1937)
*Anopheles (Anopheles) matogrosensis* Lutz and Neiva (1911)
*Anopheles (Anopheles) peryassui* Dyar and Knab (1908)
*Anopheles (Anopheles) punctimacula* Dyar and Knab (1906)
*Anopheles (Anopheles) eiseni* Coquillet (1902)
*Anopheles (Anopheles) malefactor* Dyar and Knab (1907)^b^
*Anopheles (Stethomyia) nimbus* Theobald (1902)^a^
*Anopheles (Kertezsia) cruzii* Dyar and Knab (1908)^a^
1.2. *Chagasia*
*Chagasia bonneae* Root (1927)^b^
*Chagasia ablusa* Harbach (2009)^b^
**Culicidae: 2. Subfamily Culicinae**
2.1. *Culex* (VEE vectors and West Nile virus vectors)
*Culex (Phenacomyia) corniger* Theobald (1903)
*Culex (Culex) quinquefasciatus* Say (1823)
*Culex (Culex) brevispinosus* Bonne-Wester and Bonne (1920)
*Culex (Culex) coronator* Dyar and Knab (1906)
*Culex (Culex) nigripalpus* Theobald (1901)
*Culex (Culex) pinarocampa* Dyar and Knab (1908)^a^
*Culex (Melanoconion) dunni* Dyar (1918) (VEE vector)^a^
*Culex (Melanoconion) educator* Dyar and Knab (1906)
*Culex (Melanoconion) spissipes* Theobald (1903) (VEE vector)
*Culex (Melanoconion) mistura* Komp and Rozeboom (1951)
*Culex (Carrollia) urichii* Coquillet (1906)
*Culex (Carrollia) anduzei* Lane (1944)^b^
*Culex (Lutzia) bigoti* Bellardi (1862) (predators)^a^
2.2. *Aedes* (Dengue, Mayaro, Chikungunya, VEE, and Yellow Fever vectors)
*Aedes (Stegomyia) aegypti* Linnaeus (1762) (Dengue, Chikungunya, Mayaro, and Yellow Fever vector)
*Aedes (Finlaya) terrens* Walker (1856) (potential Yellow Fever vector)
*Aedes (Ochlerotatus) scapularis* Rondani (1848) (EEV vector)
*Aedes (Ochlerotatus) serratus* Theobald (1901) (EEV vector)
*Aedes (Ochlerotatus) fulvus* Wiedemann (1828) (EEV vector)
*Aedes (Ochlerotatus) angustivittatus* Dyar and Knab (1907) (EEV vector)^a^
2.3. *Psorophora* (VEE vectors)
*Psorophora (Janthinosoma) cyanescens* Coquillet (1902)
*Psorophora (Janthinosoma) albipes* Theobald (1907)
*Psorophora (Janthinosoma) ferox* Von Humboldt (1819)
*Psorophora (Psorophora) ciliata* Fabricius (1794)^a^
*Psorophora (Psorophora) lineata* Von Humboldt (1819)
*Psorophora (Grabhamia) cingulata* Fabricius (1805)
2.4. *Mansonia* (VEE vectors)
*Mansonia (Mansonia) pseudotitillans* Theobald (1901)^a^
*Mansonia (Mansonia) titillans* Walker (1848)^a^
2.5. *Coquilletidia* (VEE vectors and West Nile virus vectors)
*Coquilletidia (Rhynchotaenia) juxtamansonia* Chagas (1907)^a^
*Coquilletidia (Rhynchotaenia) venezuelensis* Theobald (1912)^a^
*Coquilletidia (Rhynchotaenia) nigricans* Coquillet (1904)^a^
2.6. *Haemagogus* (Mayaro and Sylvain Yellow Fever vectors)
*Haemagogus anastasionis* Dyar (1921)^a^
*Haemagogus janthinomys* Dyar (1921)^a^
*Haemagogus celeste* Dyar and Núñez-Tovar (1927)
2.7. *Uranotaenia* (Avían Malaria vectors)
*Uranotaenia (Uranotaenia) typhlosomata* Dyar and Knab (1907)^a^
*Uranotaenia (Uranotaenia) calosomata* Dyar and Knab (1907)^a^
*Uranotaenia (Uranotaenia) geometrica* Theobald (1901)
*Uranotaenia (Uranotaenia) pulcherrima* Arribalzaga (1891)
*Uranotaenia (Uranotaenia) nataliae* Arribalzaga (1891)^a^
*Uranotaenia* (*Uranotaenia) leucoptera* Theobald (1907)^b^
*Uranotaenia (Uranotaenia) lowii* Theobald (1901)^a^
2.8. *Aedeomyia* (Avían Malaria vectors)
*Aedeomyia (Aedeomyia) squamipennis* Arribalzaga (1878)
**Culicidae: Subfamily Culicinae: 3. Tribe: Sabethini**
3.1. *Sabethes* (Mayaro and Sylvain Yellow Fever vectors)
*Sabethes purpureus* Theobald (1907)
3. 2. *Limatus* (potential arbovirus vectors)
*Limatus asulleptus* Theobald (1903)^a^
*Limatus durhami* Theobald (1901)
3.3. *Wyeomyia* (potential arbovirus vectors)
*Wyeomyia (Wyeomyia) celaenocephala* Dyar and Knab (1906)^a^
3.4. *Runchomyia* (facultative predators)
*Runchomyia (Ctenogoeldia) frontosa* Theobald (1903)
3.5. *Johnbelkinia* (facultative predators, arbovirus vectors)
*Johnbelkinia ulopus* Dyar and Knab (1906)^a^
3.6. *Trichoprosopon* (potential arbovirus vectors)
*Trichoprosopon digitatum* Rondani (1848)
**Culicidae: Toxorhynchitinae (agents biological control: predators)**
4.1. *Toxorhynchites* (predators)
*Toxorhynchites (Lynchiella*) *theobaldi* Dyar and Knab (1906)
*Toxorhynchites (Lynchiella*) *haemorroidalis* Fabricius (1787)^a^

*^a^New for the State*,

*^b^New for the country*.

Special attention should be placed in the new species records for Bolívar State and for Venezuela. A total of 27 mosquito species are recorded for the first time in Bolívar State. Five of them are new for the country (Tables 1 and 2) and they namely: *Anopheles (Anopheles) malefactor* Dyar and Knab (1907); *Chagasia bonneae* Root (1927); *Chagasia ablusa* Harbach (2009); *Culex* (*Carrollia*) *anduzei* Lane (1944), and *Uranotaenia leucoptera* Theobald (1907).

**Table 2:** **(A) GPS collection coordinates in localities with new records; (B) GPS collection coordinates in localities with new records**.

Localities and coordinates	Species records
**(A)**	
**SANTA ELENA**
4°36′07″61°06′34″	*Aedes angustivittatus, Culex anduzei*
4°32′53″61°08’30″	*Uranotaenia nataliae, Uranotaenia lowii*
4°36′41″61°06′22″	*Uranotaenia calosomata, Uranotaenia typhlosomata*
4°35′49″61°06′59″	*Haemagogus janthinomys, Sabethes purpureus*
4°36′01″61°06′52″	*Coquilletidia (Rhynchotaenia) venezuelensis*
**WARAMASEN**
4°33′26″61°16′59″	*Limatus asulleptus*
4°34′17″61°14′45″	*Johnbelkinia ulopus*
4°33′25″61°16′58″	*Wyeomyia (Wyeomyia) celaenocephala*
4°33′36″61°16′29″	*Anopheles (Anopheles) malefactor*
4°33′39″61°16′29″	*Uranotaenia* ***(****Uranotaenia) leucoptera*
4°33′38″61°16′28″	*Chagasia bonneae and Chagasia ablusa*
4°33′43″61°16′32″	*Culex (Melanoconion) dunni, Culex (Lutzia) bigoti*
**EL PAUJĺ**
4°28′32″61°35′34″	*Anopheles (Kertezsia) cruzii*
4°31′52″61°37′26″	*Wyeomyia (Wyeomyia) celaenocephala*
**EL PARAĺSO**
4°26′53″61°41′65″	*Anopheles (Kertezsia) cruzii*
4°26′53″61°41′65″	*Wyeomyia (Wyeomyia) celaenocephala*
**KINOK-PON**
4°33′31″61°12′47″	*Uranotaenia (Uranotaenia) leucoptera*
4°33′37″61°12′42″	*Uranotaenia (Uranotaenia) nataliae*
**WAIPARU**
4°31′52″61°37′26″	*Anopheles (Nyssorhynchus) darlingi*
**MANAKRU**
4°36′39″61°07′20″	*Toxorhynchites (Lynchiella) haemorroidalis*
4°36′24″61°07′11″	*Toxorhynchites (Lynchiella) theobaldi*
4°36′28″61°07′10″	*Coquilletidia (Rhynchotaenia) juxtamansonia*
**(B)**	
**CHIRICAYEN**
4°39′39″61°20′30″	*Chagasia bonneae and Chagasia ablusa*
4°40′10″61°20′36″	
4°42′11″61°19′48″	
4°43′03″61°19′18″	
**MAURAK**
4°33′46″61°10′46″	*Toxorhynchites (Lynchiella*) *haemorroidalis*
4°33′55″61°12′37″	*Coquilletidia (Rhynchotaenia) venezuelensis*
4°35′11″61°10′50″	*Chagasia bonneae and Chagasia ablusa*
**BETANIA**
4°39′33″61°22′59″	*Mansonia (Mansonia) titillans, Culex bigoti*
4°39′29″61°23′11″	*Psorophora (Psorophora) ciliata*
4°39′30″61°22′52″	*Mansonia (Mansonia) pseudotitillans*
4°39′25″61°22′47″	*Coquilletidia (Rhynchotaenia) juxtamansonia*
4°39′57″61°23′22″	*Coquilletidia (Rhynchotaenia) nigricans*
**SAN ANTONIO**
4°31′14″61°07′14″	*Psorophora (Psorophora) ciliata*
4°31′12″61°07′12″	*Coquilletidia (Rhynchotaenia) juxtamansonia*
4°31′13″61°07′08″	*Mansonia (Mansonia) pseudotitillans*
4°31′15″61°07′04″	*Mansonia (Mansonia) titillans*
4°31′17″61°06′56″	*Coquilletidia (Rhynchotaenia) nigricans*
4°31′16″61°06′54″	*Coqulletidia (Rhynchotaenia) venezuelensis*
**CHIRIRICA**
4°34′36″61°06′34″	*Culex (Carrollia) anduzei, Wyeomyia celaenocephala*
4°34′36″61°06′58″	*Haemagogus anastasionis, Culex (Lutzia) bigoti*
4°34′49″61°06′59″	*Haemagogus janthinomys*
4°34′49″61°11′43″	*Culex (Melanoconion) dunni*

The twenty-seven mosquito species records are namely:

1. *Anopheles cruzii* Dyar and Knab (1908).
2. *Anopheles malefactor* Dyar and Knab (1907).
3. *Anopheles nimbus* Theobald (1902).
4. *Chagasia bonneae* Root (1927).
5. *Chagasia ablusa* Harbach (2009).
6. *Culex pinarocampa* Dyar and Knab (1908).
7. *Culex anduzei* Lane (1944).
8. *Culex bigoti* Bellardi (1862).
9. *Culex dunni* Dyar (1918).
10. *Aedes angustivittatus* Dyar and Knab (1907).
11. *Mansonia titillans* Walker (1848).
12. *Mansonia pseudotitillans* Theobald (1901).
13. *Coquilletidia juxtamansonia* Chagas (1907).
14. *Coquilletidia nigricans* Coquillet (1904).
15. *Coquilletidia venezuelensis* Theobald (1912).
16. *Uranotaenia typhlosomata* Dyar and Knab (1907).
17. *Uranotaenia calosomata* Dyar and Knab (1907).
18. *Uranotaenia nataliae* Arribalzaga (1891).
19. *Uranotaenia leucoptera* Theobald (1907).
20. *Uranotaenia lowii* Theobald (1901).
21. *Psorophora ciliata* Fabricius (1794).
22. *Haemagogus anastasionis* Dyar (1921)
23. *Haemagogus janthinomys* Dyar (1921)
24. *Limatus asulleptus* Theobald (1903).
25. *Wyeomyia celaenocephala* Dyar and Knab (1906).
26. *Johnbelkinia ulopus* Dyar and Knab (1906).
27. *Toxorhynchites haemorroidalis haemorroidalis* Fabricius (1787).

## Discussion

**Figure 5:**
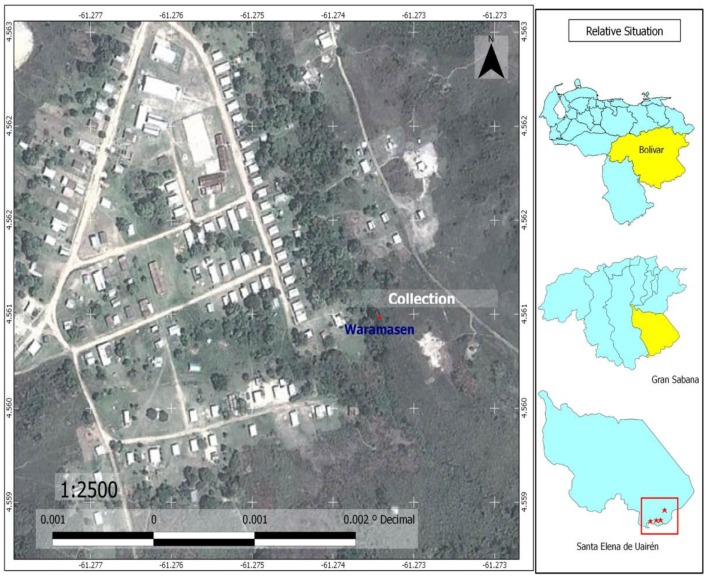
**Map of Waramasén, showing sites where larvae of *Culex bigoti, Chagasia bonneae, Chagasia ablusa, Uranotaenia leucoptera*, and *Anopheles malefactor* were collected for the first time; and showing sites where adults of *Johnbelkinia ulopus, Limatus asulleptus, Wyeomyia celaenocephala*, and *Culex dunni* were captured for the first time**.

The last revision of Anophelini Tribe (Diptera: Culicidae) in Venezuela (30) reported the total of 42 species, belonging to 2 genera: *Chagasia* (1 species) and *Anopheles* (41 species). In the present study, additional records are presented. The Anophelini species: *A. malefactor*, *Chagasia bonneae*, and *Chagasia ablusa* are three new records for Venezuela (Tables 1 and 2). These species are not potential malaria vectors. Adults and immature specimens were identified, according to keys proposed by Wilkerson (26) and Harbach and Howard (20). Larval specimens of the three species were collected in Waramasén (Figure 5) and reared to obtain associated specimens; larval habitats of the three species are streams edges, especially sites with algae and partial shade (Figure 5). The same larval habitats for *A. malefactor* and *Anopheles punctimacula* Dyar and Knab (1906) were found by Wilkerson (26). The geographical distribution of *A. malefactor* was restricted to Panamá and northwestern Colombia (26). With this new record (*A. malefactor*) their geographical distribution in Central and South America now includes Panamá, Colombia, and Venezuela. Literature records indicate that *A. punctimacula* was found naturally infected with malaria parasites in Panamá and Colombia (26). However, *A. malefactor* is not a potential malaria vector in South America (26).

**Figure 6:**
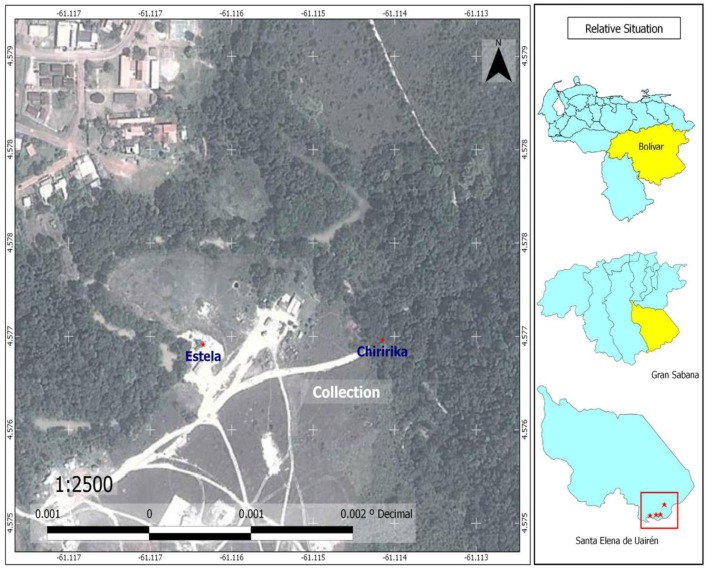
**Map of Chiririka, showing sites where adults of *Haemagogus anastasionis, Sabethes purpureus*, and *Wyeomyia celaenocephala* were captured for the first time; and showing sites where larvae of *Anopheles nimbus* (Chiririka River) and *Culex anduzei* (the Estela house) were collected for the first time in Bolívar State**.

**Figure 7:**
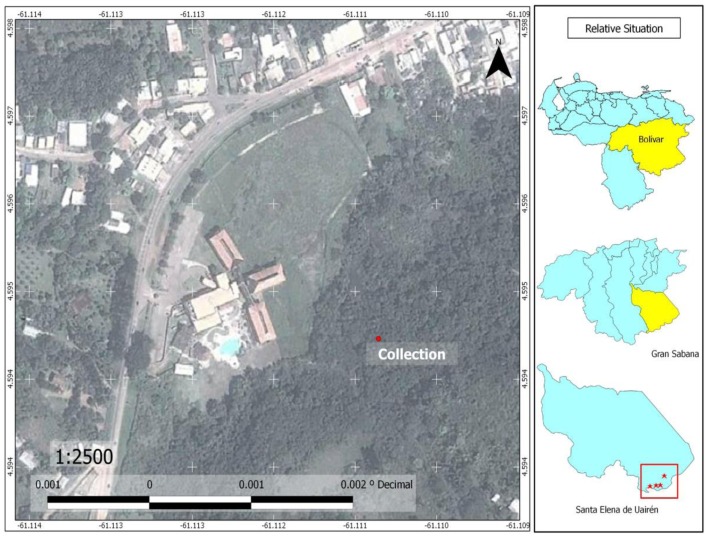
**Map of Santa Elena of Uairén, showing sites where adults specimens of *Culex (Culex) pinarocampa* and *Haemagogus janthinomys* were captured, near of the Uairén River; and showing sites where larvae of *Anopheles darlingi* were captured in pools formed near of the Uairén River bed**.

The species *Anopheles (Kertezsia) cruzii* Dyar and Knab (1908) and *Anopheles (Stethomyia*) *nimbus* Theobald (1902) are reported for the first time in Bolívar State (Table 1). Larvae of *A. nimbus* were collected in river edges of Chiririka River (Figure 6) especially in habitats with algae and partial shade. This species does not transmit diseases of medical importance to man. Adults were not collected. Larvae were reared to obtain the associated specimens. *A. nimbus* was identified, according to key proposed by Navarro (22). Females of *A. cruzii* are very aggressive crepuscular biters and they are potential malaria vectors (31); their larvae live in water of leaf axils of bromeliads (31). Adult females were captured on human landing catches, in the forest in El Paraíso and El Paují, between 16:30 and 18:45 h. Immature specimens were not collected. Diagnostic and differential characters of *A. cruzii* were confirmed in adults specimens, using descriptions of Wilkerson and Peyton in basis to 11 females collected in Iguape, Brazil (28). *Anopheles darlingi* Root (Major malaria vector in the southern Bolívar State) is reported for the first time in the Gran Sabana Municipality; also reports for the first time in South America, the two highest mosquito records for *A. darlingi*. These altitude records are: Colinas de la Laguna, with 870 MSL and Santa Elena, with 893 MSL. Larvae of *A. darlingi* were only collected in shaded pools in the forest. When the water level dropped during the dry season, pools formed in or near the river bed (Uaiparú and Uairén rivers). These localities with *A. darlingi* larval habitats are at altitudes >600 m. These altitudes can range from 600 to 1,200 m. The lower altitude record for *A. darlingi* in the Gran Sabana was found in Uaiparú River, with 628 m, and the highest altitude record was found in Santa Elena, near of the Uairén River (Figures 6 and 7) with 893 m. The same larval habitats of *A. darlingi* were found in Suriname by Rozendaal (32).

In this article, we also report for the first time in Venezuela, the presence of *Culex (Carrollia) anduzei*. With this new record, their geographical distribution in South America now includes Brazil and Venezuela. Immature specimens were identified, according to keys proposed by Valencia (25). *C. anduzei* females do not transmit diseases of medical importance to man (25). Larvae were collected in plastic and metallic artificial containers in Chiririka (Figure 6), El Paraíso, El Paují, and Santa Elena (Figure 7). Adult specimens of *C. anduzei* were not captured. Additionally, we report for the first time the presence of *Culex* (*Lutzia*) *bigoti* Bellardi in Bolívar State. This species does not transmit diseases of medical importance to man. Larval specimens were collected in Waramasén and were found in artificial containers (tires, and plastic and metallic containers). It is well known that *Cx. bigoti* larvae are predators in several artificial containers (tires, tin cans, old paint cans, plastic and metallic containers). In Paraná State, Brazil, larval specimens of *C. bigoti* were found in tires, cans, and cut bamboo (33). In Venezuela, this species was collected in artificial containers (tires and plastic containers) but, was not collected in epiphytic and terrestrial bromeliads (12, 34). *Toxorhynchites* (*Lynchiella*) *theobaldi* Dyar and Knab (1906) and *Toxorhynchites* (*Lynchiella*) *haemorroidalis* Fabricius (1787) were collected in tires and tree-holes; they are a potential predators used for mosquito control. A long-term investigation in Florida (USA), demonstrated a reduction in tree-holes, mosquitoes, attributable to predation by *Toxorhynchites rutilus* (35).

Additionally, *Culex dunni* Dyar (1918); *Aedes* (*Ochlerotatus*) *angustivittatus* Dyar and Knab (1907); and *Psorophora* (*Psorophora*) *ciliata* Fabricius (1794) are reported for the first time in Bolívar State. These three species are potential vectors of the VEE (36–38). In addition, we reports three new species of the genus *Coquilletidia* and two new species of *Mansonia* for Bolívar State; they are namely: *Mansonia titillans* Walker (1848), *Mansonia pseudotitillans* Theobald (1901), *Coquilletidia juxtamansonia* Chagas (1907), *Coquilletidia venezuelensis* Theobald (1912), and *Coquilletidia nigricans* Coquillet (1904). The two species of the genus *Mansonia* are potential vectors of the epizootic cycle of the VEE (36) and *Coquilletidia venezuelensis* is a potential vector of enzootic cycle of the West Nile virus in Venezuela (39).

The genus *Haemagogus* Williston includes mosquitoes with diurnal activity and immature habitats on Phytotelmata (tree-holes and cut bamboo internodes). Adult females bite in forests during the day (40). *Haemagogus* species have been involved in yellow fever transmission, a virus circulating in forest areas in Latin America among arboreal primates and marsupials by means of mosquito bite (40). The genus comprises 28 species; 9 of them are present in Venezuela (4). The presence of *Haemagogus anastasionis* Dyar (1921), *Haemagogus janthinomys* Dyar (1921), and *Haemagogus celeste* Núñez-Tovar (1927) was detected in forest areas of Chiririka and Santa Elena (Figures 6 and 7). Adult specimens were identified, according to photographical key proposed by Liria and Navarro (4). These three species were captured on human landing catches in the forest, between 16:30 and 18:45 h, near of the Uairén and Chiririka rivers edges (Figures 6 and 7). Immature specimens were not collected. *H. anastasionis* and *H. janthinomys* species are two potential vectors of jungle yellow fever and jungle cycle of Mayaro virus in Venezuela (4); and both are new species records for the Gran Sabana and the Bolívar State. With these two new records, the genus *Haemagogus* in Bolívar State now includes six species: *H. celeste* Dyar & Núñez-Tovar, *H. equinus* Theobald, *H. leucocelaenus* (Dyar & Shannon), *H. albomaculatus* Theobald, *H. anastasionis* Dyar, and *H. janthinomys* Dyar. The species *Sabethes purpureus* Theobald (1907) was collected on human landing catches near of the Uairén River edge (Figure 7). This species is also a potential vector of sylvatic yellow fever and jungle cycle of Mayaro virus. The Mayaro alpha virus produces non-specific, sub-lethal disease symptoms, often confused with dengue, but with symptoms of arthralgias (arthrosis) that can cause incapacitating disability (40). The Mayaro virus shows a great plasticity in vertebrate host infection, whereas high specificity in the mosquitoes of Culicidae family, vectors in the jungle cycle. Risk factors of infection are associated with forest areas of northern South America and the rainy season (40). The enzootic cycle is similar to the jungle cycle of yellow fever, which involves *Haemagogus* mosquitoes and monkeys as reservoirs (40). However, the involvement of others secondary vectors (e.g., *Sabethes*) and others hosts may be important in spread of the virus. Humans may have high levels of viremia and efficient experimental transmission has been demonstrated in *Aedes aegypti*, *Aedes albopictus*, and *Aedes scapularis*, suggesting a significant risk to public health in urban, rural, and domestic locations close to enzootic foci of Mayaro virus (40). In addition, we collected adults of *A. aegypti* and *A. scapularis* Rondani (1848) in urban and periurban areas of Santa Elena, Waramasén, Chiririka and Maurak, representing a significant risk to the inhabitant’s populations of theses localities. *A. albopictus* specimens were not collected in the Gran Sabana.

*Aedes aegypti* and *A. albopictus* species have been involved in dengue transmission in the Manaus rural areas, Brazilian Amazon (3). The presence of *A. albopictus* in the Brazilian Amazon represents a potential risk of transmission of Chikungunya and Mayaro virus in urban, rural, domestic, and wild environments (1). The occurrence of its larvae and pupae, breeding in the same containers with other domestic species, associated with several sources of blood meal available in urban, rural, and wild environments, reveal its gradual establishment in the indoor of households and its potential involvement in the transmission of zoonotic pathogens to humans. The ability that their eggs may remain viable in nature for long periods of diapauses and the demonstrated transovarial transmission occurrence of several arboviruses has raised the need to expand the strategies directed toward combating *A. albopictus* in Vector Control Programs in South American (1). In Venezuela, *A. albopictus* was detected for the first time in 2009 (41), suggesting a significant risk to public health in urban, rural, domestic, and wild environments (1). Dormancy of the egg stage (and drought resistance) is considered to be a reproductive strategy for the long-term survival of multivoltine mosquitoes that develop in temporary habitats, such as tree-holes and other natural water containers that are subject to water fluctuations (42). Egg diapause involves a long stable arrest of hatching, even when environmental conditions are favorable for hatching (42).

Additionally, in this article we also report for the first time three new records of *Sabethini* Tribe mosquitoes for the Bolívar State (Table 1); and they are: *Wyeomyia celaenocephala* Dyar and Knab (1906), *Limatus asulleptus* Theobald (1903), and *Johnbelkinia ulopus* Dyar and Knab (1906). Adult females were collected on human landing catch during the day in Waramasén and Chiririka (Figures 5 and 6). These three species are potential arboviruses vectors in Venezuela (43) and *J. ulopus* larvae are facultative predators (43).

Species of the genus *Runchomyia* (Table 1) does not transmit diseases of medical importance to man; their larvae live in leaf axils of epiphytic and terrestrial bromeliads and in floral bracts (19). In 1986, the species *Runchomyia frontosa* Theobald was reported in carnivorous bromeliads from the Gran Sabana, Venezuela. The immature mosquitoes were collected living in the fluids held by the carnivorous bromeliad *Brocchinia reducta* Baker (44). The larvae of *Runchomyia frontosa* is a facultative predator, filter feeding, or consuming large prey that it captures with its enlarged maxillae in carnivorous bromeliads (44). Adult females of *Runchomyia frontosa* were collected on human landing catch during the day in El Paraíso and Waramasén (Table 1). This species does not transmit diseases of medical importance to man (19).

In addition we report, five new records of the genus *Uranotaenia* in Bolívar State, they are: *Uranotaenia typhlosomata* Dyar and Knab (1907); *Uranotaenia calosomata* Dyar and Knab (1907); *Uranotaenia nataliae* Arribalzaga (1891); *Uranotaenia leucoptera* Theobald (1907); and *Uranotaenia lowii* Theobald (1901). Species of genus *Uranotaenia* were found mainly in ground-water habitats, including springs, stream margins, herbaceous swamps, and temporary pools with vegetation (19). Some species have been found in tree-holes, plant axils, and artificial containers. Females of some species are known to feed on frogs, birds, and mammals, but are normally not attracted to humans (19). They are avian malaria vectors in Venezuela. In the Llanos of Venezuela, was found a high endemicity of avian malaria (45, 46). Immature specimens were collected in lagoons and herbaceous swamps (Morichales) in Santa Elena de Uairén, Waramasén, Maurak, Colinas de la Laguna, Altamira, San Antonio, Kinok-Pon-Parú, La Primavera, Chiricayén, Manak-Krú, Maurak, and Uaiparú. *Uranotaenia leucoptera* was collected only in tree-holes in Waramasén and Manak-Krú. This species is recorded for the first time from Venezuela. This study also extends the geographical distributions of *Uranotaenia leucoptera* in South America to Venezuela.

These findings shows the importance of further studies related to mosquito’s geographical distribution, ecological aspects, arbovirus detection, epidemiological surveillance, and possible epidemiological link with emerging and reemerging arboviruses in the common border of Brazil and Venezuela. The entomological surveillance has an important role among the preventive measures against emerging diseases transmitted by insects, particularly by mosquitoes.

## Conflict of Interest Statement

The authors declare that the research was conducted in the absence of any commercial or financial relationships that could be construed as a potential conflict of interest.
